# A semi-automated method using object-based image analysis (OBIA) to detect and enumerate beluga whales in summer from very high-resolution (VHR) satellite imagery

**DOI:** 10.1371/journal.pone.0307716

**Published:** 2024-11-13

**Authors:** John Iacozza, Bryanna Sherbo, Cortney Watt

**Affiliations:** 1 Fisheries and Oceans Canada, Freshwater Institute, Winnipeg, MB, Canada; 2 Department of Environment and Geography, University of Manitoba, Winnipeg, MB, Canada; Bielefeld University: Universitat Bielefeld, GERMANY

## Abstract

Very high-resolution (VHR) satellite imagery has proven to be useful for detection of large to medium cetaceans, such as odontocetes and offers some significant advantages over traditional detection methods. However, the significant time investment needed to manually read satellite imagery is currently a limiting factor to use this method across large open ocean regions. The objective of this study is to develop a semi-automated detection method using object-based image analysis to identify beluga whales (*Delphinapterus leucas)* in open water (summer) ocean conditions in the Arctic using panchromatic WorldView-3 satellite imagery and compare the detection time between human read and algorithm detected imagery. The false negative rate, false positive rate, and automated count deviation were used to assess the accuracy and reliability of various algorithms for reading training and test imagery. The best algorithm, which used spectral mean and texture variance attributes, detected no false positives and the false negative rate was low (<4%). This algorithm was able to accurately and reliably identify all the whales detected by experienced readers in the ice-free panchromatic image. The autodetection algorithm does have difficulty separately identifying whales that are perpendicular to one another, whales below the surface, and may use multiple segments to define a whale. As a result, for determining counts of whales, a reader should manually review the automated results. However, object-based image analysis offers a viable solution for processing large amounts of satellite imagery for detecting medium-sized beluga whales while eliminating all areas of the imagery which are whale-free. This algorithm could be adapted for detecting other cetaceans in ice-free water.

## Introduction

Beluga whales (*Delphinapterus leucas*) have a circumpolar Arctic and sub-Arctic distribution [1]. They are medium-sized toothed whales with a body length greater than 3 m for adults. The adults are iconically known for their white coloration, which allows them to camouflage in ice, but can result in a stark difference between them and their environment in the summer ice-free period [2]. Beluga whale distribution changes seasonally because of annual formation and melting of sea ice, and thus they undertake, in some cases, extensive migrations to ice free areas in the winter months and return to estuaries where they are distributed in the summer ice-free season, with only a few populations known to reside in the same geographic region year-round [3]. Aside from sea ice, beluga whale preferred habitat also depends on a number of factors including temperature, river discharge, tidal cycles, and bottom bathymetry [4]. Beluga whales generally have site fidelity to natal estuaries and low dispersal rates [5]. As a result, surveys for estimating population abundance of beluga whales are often undertaken in estuaries in the summer since the beluga whales congregate at this time [6].

Currently beluga whale population abundance is primarily estimated using aerial survey methods with visual observers or cameras to capture continuous photographs [7–9]. These surveys require a team of people (four visual surveyors, a camera operator, co-pilot and pilot) and can take weeks to cover the distributional range of the whales. These surveys are often time limited due to whale migratory behavior, inhibited by poor weather, and can cost hundreds of thousands of dollars to conduct. Surveys designed with systematic transect lines can also not account for whale movement into and out of the transect strip. Typically, this is addressed by assuming whale movement is random; however, social structure and communication can result in coordinated movements [10, 11]. With the availability of very high-resolution (VHR) optical satellite imagery in the late 1990s/early 2000s, the question has been raised of whether imagery with a fine spatial resolution (less than one m) can be used to estimate marine mammal distribution in remote areas such as the open ocean and in the Arctic. Satellite imagery offers a potential alternative to aerial surveys and can overcome some survey challenges. For instance, because the satellite image covers a vast swath of the ocean surface in a single pass, a distribution of whales in open water can be estimated without having to assume random whale movement. Additionally, satellites can image offshore areas of the ocean that are inaccessible to aerial surveys, thus able to provide a more accurate assessment of the distribution of whales.

Studies have used VHR to visually identify marine mammal and bird species, including Wandering Albatross *(Diomedea exulans)* at South Georgia and Northern Royal Albatross (*Diomedea sanfordi*) on the Chatham Islands [12], Weddell seals *(Leptonychotes weddellii)* in Antarctic [13], beluga whales in the Eastern Canadian Arctic [14] and baleen whales in the Western Antarctic Peninsula [15]. Satellite technology has the possibility of imaging large areas at fine spatial resolutions; however, the time required to visually read the satellite imagery is currently a significant limitation for using this method on a large scale. While in theory, satellite imagery can be used to estimate whale population abundance and density, this method can be time and labour intensive, depending on the geographical coverage of the study area, and require technical expertise. Human fatigue can also negatively affect detection accuracy. Crowdsourcing reduces the overall time required to identify features of interest but requires a large number of volunteers who may have limited experience, which can reduce detection accuracy. Another limitation with crowdsourcing is it may need to be led by the imagery supplier as the licensing agreements often restrict sharing the imagery with the public to detect the whales.

Automated detection methods have been developed to identify features of interest in VHR remote sensing imagery. These methods reduce the time required to identify the objects and potentially reduce error related to human fatigue. The detection methods can be divided into two groups: pixel-based algorithms and semi-automated object-based image analysis (OBIA). Pixel-based methods (e.g., supervised classification and thresholding) classify individual pixels into discrete classes based on the spectral information. Cubaynes et al. [16] used spectral image analysis of WorldView-3 imagery (with a spatial resolution of 31 cm) to identify four mysticete species in various oceans. Fretwell et al. [17] demonstrated that classification methods using the spectral information can be used to classify Southern right whales *(Eubalaena australis)* in the waters off of Argentina in VHR satellite imagery. One limitation of using only spectral information is that other features (e.g., sea state) can have similar spectral signatures as the whale in optical imagery, leading to misidentification and thus lower accuracy in the final classification. Additionally, whales slightly below the surface can appear spectrally very similar to the surrounding water and thus, may not be identified as discrete objects.

Object-based image analysis is relatively new and less developed than pixel-based algorithms. These methods first segment features in the image into homogenous objects and then categorize these objects into discrete classes using spectral, spatial and/or textural attributes. This method has been successfully used in urban areas to identify infrastructure, such as roads, cars and buildings [e.g. 18–20]. More recently this method has been successfully used to identify wildlife, including, terrestrial marine mammals [21], bush-tailed penguin (*Pygoscelis* ssp.) guano in Antarctica [22], and lesser snow geese *(Chen caerulescens caerulescens)* in aerial photos [23]. The accuracy of the classification is based on the image segmentation, which may be problematic for small features in the imagery [24, 25]. With the availability of sub-50 cm resolution imagery, beluga whales are visible in the imagery and object-based image analysis methods should be able to identify these features in Arctic waters.

The objective of this study is to develop a semi-automated method using OBIA to identify beluga whales in open water (summer) ocean conditions in the Arctic using WorldView-3 satellite imagery. We also assess the efficiency in time of using this method, compared to visual manual detection of whales in the imagery.

## Methods

### Description of satellite imagery

**Fig 1:**
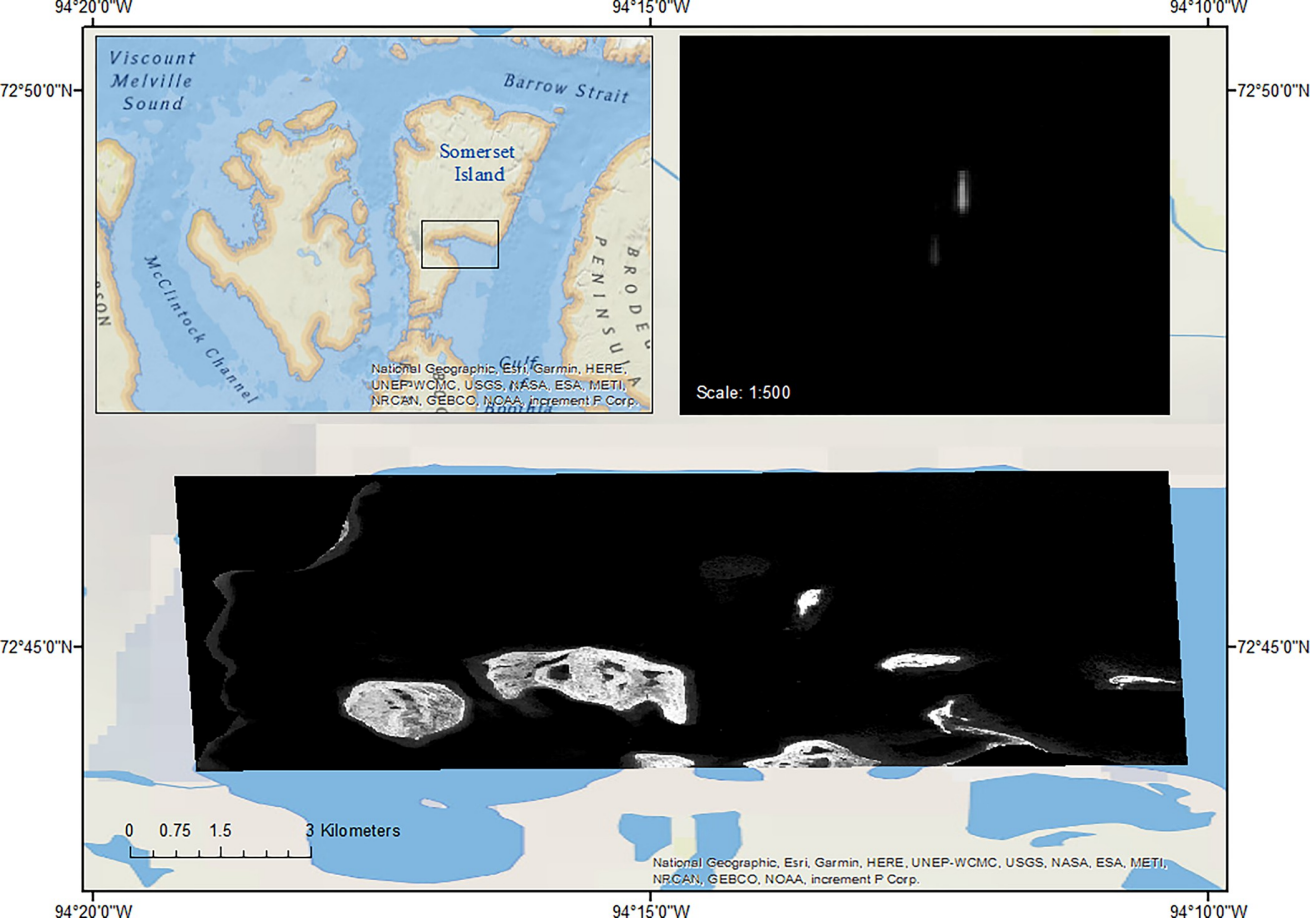
Location of study area in the eastern Canadian Arctic (inset) with a panchromatic WorldView-3 satellite image (catid: 1040010061AFA400) used in this study to develop an algorithm to semi-automate identification of beluga whales. The top right inset shows an example of a beluga whale and calf at a scale of 1:500. (Reprinted under a CC BY license, with permission from Maxar Technologies, original copyright 2020).

A VHR panchromatic WorldView-3 image (catid: 1040010061AFA400) was acquired on August 05, 2020 for Creswell Bay (centered at 72°45’N, 94°14’W), along the eastern coast of Somerset Island in northern Canada (Fig 1). The image was provided by Maxar Technologies. The study area consisted of the summer core habitat for the Eastern High Arctic–Baffin Bay beluga whale population [26, 27]. The level 2 georeferenced image consisted of a single panchromatic band (450 to 800 nm), with a spatial resolution of 31 cm and a spatial extent of approximately 24.2 km^2^. The grey-scale image was cloud- and sea ice-free with a low Beaufort state, and multiple islands located in the lower half of the image (Fig 1). Beluga whales are clearly visible in the image at a scale of 1:500 (Fig 1 inset).

The image was opened in ENVI© v.5.5 image processing software (Excelis Visual Information Solutions, Boulder, CO, USA) using a laptop computer with an Intel processor (i7-9850H), 2.60 GHz and 32 GB of RAM. To reduce the time required to process the imagery and conduct subsequent image analysis, the original image was divided into 9 separate subsets or tiles in ENVI©. Each tile covered an area of approximately 1638.3 m x 1638.3 m and were 58 MB in size. The algorithms were developed on the 20AUG05_R3C2 tile (referred to as the training tile) and tested on a second tile, 20AUG05_R2C3 (referred to as the test file). A third tile, 20AUG05_R2C1, (referred to as the no-whale tile) contained no visible whales and was used to test the reliability of the method in identifying beluga whales.

### Image pre-processing

**Fig 2:**
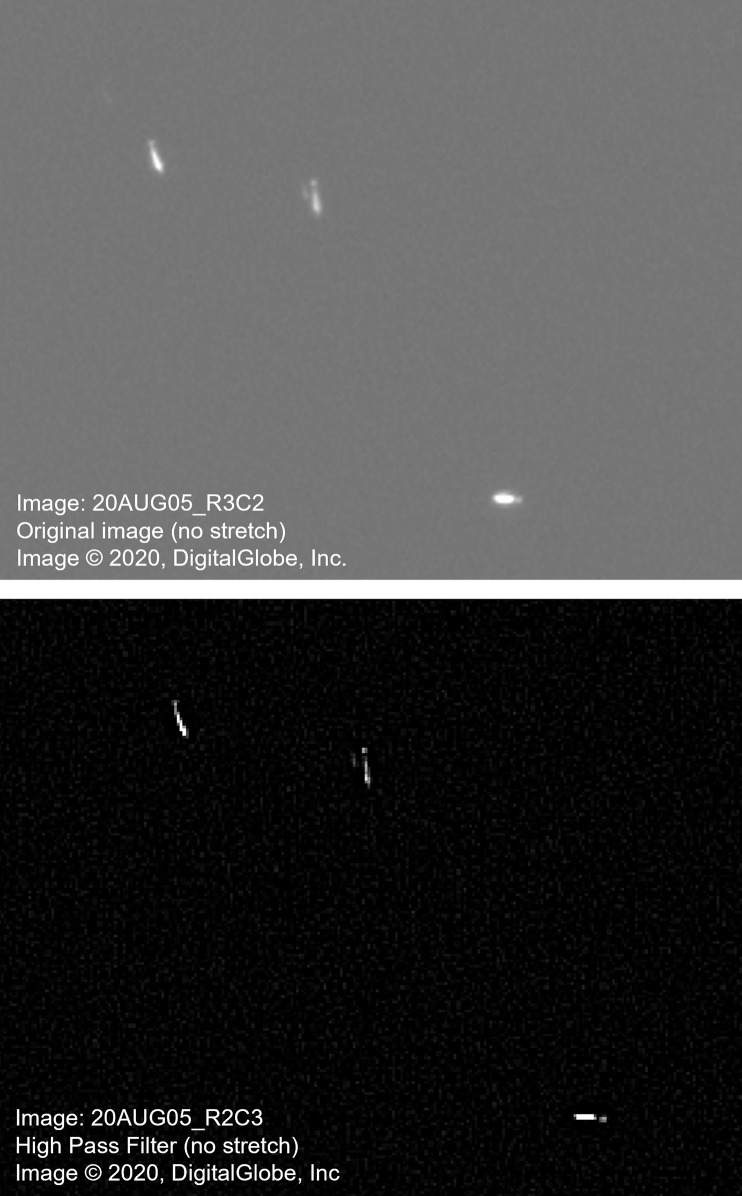
Examples of (a) the original panchromatic WorldView-3 satellite image (no stretch) and (b) the same tile of the satellite image after a high-pass filter was applied. Both images are at a scale of 1:250. (Reprinted under a CC BY license, with permission from Maxar Technologies, original copyright 2020).

The initial step in the pre-processing of the imagery involved masking any land present in the image. Regions of interest (ROIs) that covered land were manually digitized in ENVI© v.5.5. The next step was to apply a high-pass spatial filter to the land masked image. This filter extracted the high-frequency information (i.e. whales) in the image, while deemphasizing the low frequency information (i.e. open water). This is a local filter, in which the central pixel value in a moving window is modified based on the spectral value in neighbouring pixels. For this study, the smallest window size (3x3) was selected for the high-pass filter in order to retain most of the high spatial resolution of the original image. The resultant image highlighted the whales and minimized the noise in the surrounding water that may impact the precision and accuracy of the semi-automated detection (Fig 2).

### Semi-automated detection method

**Fig 3:**
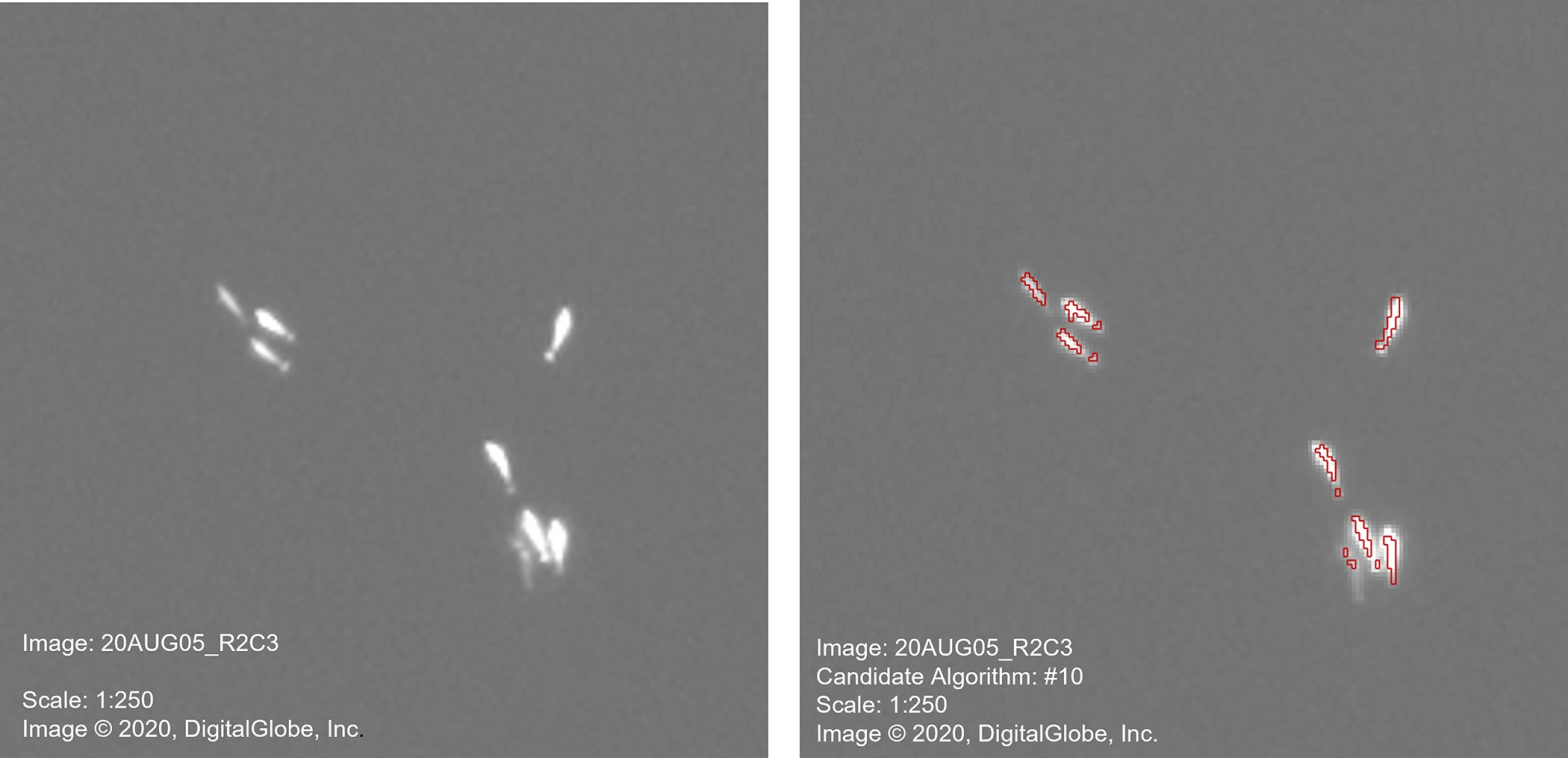
Example of image segmentation. The image on the left represents the original image showing the location of the whales, while the image on the right illustrates the segments produced by the OBIA method (using an EDGE segment algorithm with a scale level of 90) and a FAST LAMBDA merge algorithm with a merge level of 40). (Reprinted under a CC BY license, with permission from Maxar Technologies, original copyright 2020).

Object-based image analysis (OBIA) was used to identify the location of the beluga whales from the high-pass filtered, land-masked panchromatic image. This was accomplished using the Rule-Based Feature Extraction module in ENVI©. The first step in OBIA is segmentation, which creates segments in the image by grouping neighbouring pixels with common spectral, spatial and/or texture values. Theoretically, these segments would correspond to the whales, or parts of the whale that were distinct from the water surface (Fig 3). In ENVI©, a watershed algorithm is used for segmentation, which transforms the image into a contrast gradient map with higher pixel values representing the boundaries between the whales and the open water [28]. The choice of segmentation algorithm includes *EDGE*, which is best for detecting edges of features with sharp boundaries (e.g., ‘lighter’ spectral whales against ‘darker’ water) and *INTENSITY*, which is best for images with subtle gradients in the spectral values (e.g., digital elevation models). The *EDGE segment algorithm* was used in this study, as whales in open water have sharp boundaries with the open water. The amount of segmentation can be adjusted, with high levels resulting in fewer segments, and low levels producing more segments. For this study, various levels were chosen and tested on the training tile.

Next, merging is used to combine neighboring segments with similar values. This is accomplished by selecting a merge algorithm and merge level. Merging can use either the *FULL LAMBDA SCHEDULE*, in which smaller segments are merged with larger areas, or *FAST LAMBDA*, in which adjacent segments are merged with segments within similar spectral levels and border sizes [29, 30]. For this study, the *FAST LAMBDA* merge algorithm was selected since the beluga whale segments were spectrally more similar than the surrounding water. The level of merging can be adjusted, with larger values resulting in more merging of the segments. Again, a variety of merge levels were tested on the training tile to determine which would be appropriate for identifying beluga whales in this image.

Finally, a kernel size is selected. The kernel is a convolution matrix that is used to calculate the texture values used in segmentation, as well as the attributes used to assign the segments to classes (as described below). The size of the kernel represents the pixels in the moving window used to compute the texture attributes. Kernel sizes are odd numbers varying from three to 19 pixels. Because the whales are not large objects in the original images, a kernel size of three was selected for all algorithms tested on the training tile.

The second step in OBIA is object identification, which uses rules to assignment the segment into information classes. These rules are based on the selection of one or more attributes that are used to define the feature (i.e., whales). These attributes that can be used included four spectral attributes, which compare the spectral value for a particular segment, four texture attributes (calculated using the kernel size), and 14 spatial attributes, which are computed for the segment (S1 Table contains a list of the spectral, textural and spatial attributes available). We used various combinations of the attributes, along with scale and merge levels, on the test image to determine which combination is best for identifying beluga whales. A preview window, available in the software package, was used to visually assess the ability of the various levels and attributes in assigning the beluga whale class in the segmented training tile. This study then tested the eleven ‘best’ algorithms (referred to as the candidate algorithms, or CA) on the test tile and the no-whale tile to determine which was most reliable and accurate at identifying beluga whales in open water.

### Visual identification and accuracy assessments

Image tiles were examined by two experienced observers at a scale of 1:800, and each feature of interest was identified by adding a georeferenced point. Features were identified by their whale-like size and shape, between one to 4.5 m in length and roughly oval shaped. Many whales were swimming in groups and some whales were identified due to their close proximity to groups, these points included whales under the surface or smaller, potentially younger whales. Each feature point was given a confidence rating: two for an obvious whale (right shape and size) or one for a probable whale (approximate shape and size). An experienced observer double checked all whale points. The locations of each whale were digitized manually and the locations on each tile were saved as a GIS shapefile and compared to the locations identified by each candidate algorithm. In addition, the time required to identify the beluga whales in each tile was recorded by the observer. This was compared to the time required to run the candidate algorithms on each tile.

To assess the accuracy and reliability of the algorithms proposed for the semi-automated whale detection, whales in the training and test tiles were visually identified by two experienced observers. These observers were independent of the image analysis and not involved in the development of the detection algorithms. The whales identified by both observers were considered as verifiable or ‘true’ whale locations. The ‘true’ locations were then compared to the objects identified by each algorithm. True positives (TP) were defined as objects identified as a whale by both the candidate algorithm and by the observer. False positives (FP) were objects identified as a whale by the candidate algorithm but not the observers and represent an overcount, while false negatives (FN) were objects identified as a whale by the observer but not the candidate algorithm and are an undercount.

Several metrics, including the false negative rate (FNR), false positive rate (FPR) and automated count deviation (ACD) (following [31]), were calculated based on these counts. These metrics were used to assess the accuracy and reliability of each algorithm for both the training and test tiles and provide a more direct assessment of the accuracy and reliability of the semi-automated detection algorithm [31]. The false negative rate (or missed rate) provides a direct measure of the undercount for each algorithm. It is calculated as a ratio between the number of false negatives and the total number of whales manually identified by the observer:

$$FNR=\frac{FN}{identified\;whales}x\;100\%$$ [1]

The false positive rate is a measure of the accuracy of the algorithm. It is calculated as the ratio between the number of false positives and the total number of whales manually identified by the observer:

$$FPR=\frac{FP}{identified\;whales}x\;100\%$$ [2]

The automated count deviation is a measure combining the false negative and false positive rates, providing a single measure of the accuracy and reliability of the algorithm. It is calculated using:

$$ACD=\frac{FP+FN}{identified\;whales}x\;100\%$$ [3]

All three of these metrics range between 0 and 100, with values closer to 0 indicating a more accurate and reliable detection of whales.

## Results

The eleven ‘best’ candidate algorithms for identifying beluga whales in the VHR satellite imagery are listed in Table 1. Initial examination involved the determination of best scale and merge levels used for segmentation of the objects in the satellite imagery. This was done using the segmentation previewing function in the Rule-Based Feature Extraction module in ENVI©. Most of the candidate algorithms used a high scale value (80 or 90; Table 1). There was more variation in the optimal merge levels, with most applying a moderate merge level of 40 (Table 1). Changing either the scale or merge levels in small increments (less than ten), when holding the other level constant, did not produce any significant changes in the segmentation. Three attributes, including spectral mean (mean value of the pixels comprising the object in the high-pass filter image), texture entropy (average spectral intensity distribution of the pixels in the kernel) and texture variance (the average variance of the spectral value in the kernel), used either alone or in combination, produced the best results for identifying the segments as whales (Table 1). In the high-pass filtered image, the spectral mean ranged from -660 to 920 and a value greater than 35 was optimal for identifying whales in the image. In terms of texture variance, the values in the image ranged from 750 to 500,000, and a value greater than 1,500 was used in some of the candidate algorithms to classify the objects as whales. The texture entropy ranged from -2.25 to -0.11 for the image, and the OBIA algorithms used a range of between -0.80 to -0.40 (Table 1). The inclusion of spatial attributes in the rules for the algorithms, such as elongation, major length and area (either alone or in combination with other spectral and/or texture attributes) did not improve the classification of the segments.

**Table 1:** The eleven ‘best’ candidate OBIA algorithms used in this study to identify beluga whale locations. The segment algorithm was EDGE and the merge algorithm was FAST LAMBDA for all of the eleven algorithms listed.

*Algorithm*	*Scale Level*	*Merge Level*	*Attribute(s)*
*CA#1*	*80*	*40*	*Spectral mean (>40)*
*CA#2*	*80*	*70*	*Spectral mean (>40)*
*CA#3*	*80*	*20*	*Spectral mean (>40)*
*CA#4*	*30*	*10*	*Spectral mean (>60) and Texture Entropy (-0*.*80 to -0*.*44)*
*CA#5*	*90*	*40*	*Spectral mean (>50) and Texture Entropy (-0*.*80 to -0*.*44)*
*CA#6*	*90*	*40*	*Spectral mean (>45) and Texture Entropy (-0*.*76 to -0*.*40)*
*CA#7*	*90*	*40*	*Spectral mean (>45) and Texture Entropy (-0*.*80 to -0*.*44)*
*CA#8*	*90*	*10*	*Spectral mean (>45) and Texture Entropy (-0*.*76 to -0*.*40)*
*CA#9*	*90*	*40*	*Spectral mean (>35) and Texture Variance (>2000)*
*CA#10*	*90*	*40*	*Spectral mean (>35) and Texture Variance (>1500)*
*CA#11*	*90*	*40*	*Spectral mean (>35) and Texture Variance (>1740)*

**Fig 4:**
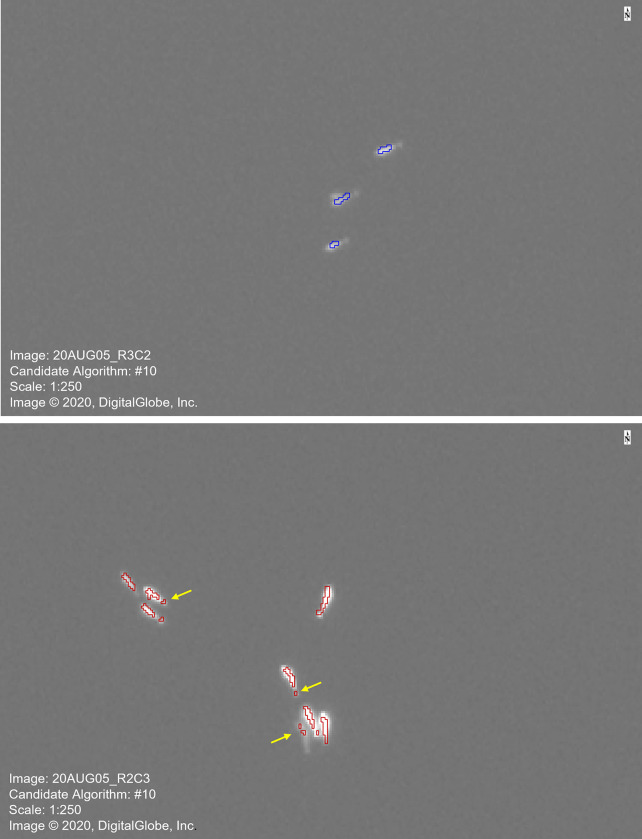
Examples of OBIA of beluga whales for two tiles from the original WorldView-3 satellite image. Both images are at a scale of 1:250. Arrows indicate multiple segments of the same whale. (Reprinted under a CC BY license, with permission from Maxar Technologies, original copyright 2020).

The outputs from the candidate algorithms were compared to the location and number of whales identified by the experienced observer and accuracy metrics were calculated (Table 2). Using the training tile, ACD and FNRs were lowest for algorithms that incorporated texture variance attributes (CA#9, #10 and #11) into the classification of the objects. Using the test tile, the FPRs were low (between 0% and 3.16%) for all algorithms, and there was a reduction in FPR from the training tile (between 3.13% and 46.88%). In addition, the ACDs decreased for most of the algorithms, when comparing the results of the training and test tiles. Overall, CA#10 had the lowest FPR, FNR and ACD index for both training and test tiles (Table 2), indicating the use of spectral mean and texture variance attributes were able to accurately and reliably identify all the whales detected by experienced readers in the ice-free panchromatic image. Fig 4 illustrates the identification of beluga in the training tile (top image) and test tile (bottom image) using CA#10.

**Table 2:** Accuracy assessment matrix for the eleven candidate algorithms for the training tile (20AUG05_R3C2) and the test tile (20AUG05_R2C3). Observer #1 visually identified a total of 32 whales in 20AUG05_R3C2 and 190 in 20AUG05_R2C3, while observer #2 detected 34 and 193 whales respectively. The total number of identified whales (total count) are those identified by both observers. The time required to identify the whales in each image is included in the last column (average time for the candidate algorithms).

*Image*: *20AUG05_R3C2 (training tile) Total Number of Identified Whales*: *32*							
*Algorithm*	*Total Count*	*False Positive*	*False Negative*	*False Negative Rate (%)*	*False Positive Rate (%)*	*Automated* *Count* *Deviation (%)*	*Average Time (mins)*
*CA#1*	45	15	2	6.25	46.88	53.13	
*CA#2*	41	12	3	9.38	37.50	46.88	
*CA#3*	47	16	1	3.13	50.00	53.13	
*CA#4*	28	1	5	15.63	3.13	18.75	
*CA#5*	27	0	5	15.63	0.00	15.63	
*CA#6*	28	0	4	12.50	0.00	12.50	
*CA#7*	33	5	4	12.50	15.63	28.13	
*CA#8*	33	5	4	12.50	15.63	28.13	
*CA#9*	33	1	0	0.00	3.13	3.13	
*CA#10*	32	0	0	0.00	0.00	0.00	
*CA#11*	32	0	0	0.00	0.00	0.00	
							3:42
*Observer #1*	32						14:26
*Observer #2*	34						28:00
*Image*: *20AUG05_R2C3 (test tile) Total Number of Identified Whales*: *190*							
*Algorithm*	*Total Count*	*False Positive*	*False Negative*	*False Negative Rate (%)*	*False Positive Rate (%)*	*Automated* *Count* *Deviation (%)*	*Average Time (mins)*
*CA#1*	193	6	3	1.58	3.16	4.74	
*CA#2*	167	0	23	12.11	0.00	12.11	
*CA#3*	190	3	3	1.58	1.58	3.16	
*CA#4*	172	0	18	9.47	0.00	9.47	
*CA#5*	176	0	14	7.37	0.00	7.37	
*CA#6*	171	0	19	10.00	0.00	10.00	
*CA#7*	176	0	14	7.37	0.00	7.37	
*CA#8*	180	0	10	5.26	0.00	5.26	
*CA#9*	175	0	15	7.89	0.00	7.89	
*CA#10*	184	0	6	3.16	0.00	3.16	
*CA#11*	180	0	10	5.26	0.00	5.26	
							4:46
*Observer #1*	190						31:02
*Observer #2*	193						30:00

The candidate algorithms were also run on the no-whale tile. The first three CAs, which only used the spectral mean, had false positives, while all other CAs accurately identified no whales in the tile. None of the candidate algorithms identified any false negatives in this tile.

The experienced observer (#1) identified 32 whales in the training tile (in a total of 14:26 minutes) and 190 whales in the test tile (in 31:02 minutes) (Table 2). Experienced observer #2 identified 34 whales in the training tile (in a total of 28:00 minutes) and 193 whales in the test tile (in 30:00 minutes) (Table 2). There was little variability between observers in terms of counts (two and three whales in the training and test tiles respectively), and locations. Manual observation ranged from 5:32 to 11:56 minutes per one km^2^ for both observers and tiles at a at a scale of less than 1:800. The time required to identify beluga whales on either tile using the candidate algorithms averaged 4:02 minutes with no significant difference in time required to run the candidate algorithms (standard deviation = 32 secs). Of the total whale count, 7.2% (n = 15) were undetected by observer #1, and 0% (n = 0) were undetected by observer #2.

## Discussion

This research focused on the utility of object-based image analysis for identification of beluga whales in ice-free conditions in the Arctic. The method developed was able to identify beluga whales using a semi-automated technique which was more efficient than visual identification; however, a human observer is still required to count the whales. Previous studies have used mainly spectral-only pixel-based classification methods to create an automated detection algorithm for whales (e.g., [17, 16]). This current method focused on using an object-based analysis technique which offers several advantages over pixel-based classification. Spectral only pixel-based analyses do not take into consideration other attributes like shape, size, color, compactness, and association, which are useful when characterizing features as whales. On the other hand, OBIA changes the classification units from pixels to image objects which reduces within-class spectral variation and can integrate a large set of features characterizing an objects spatial, textural, and contextual properties to improve detection accuracy [32, 33].

The optimal algorithm used a high scale level (90) and moderate merge level (40) in the segmentation of the image (Table 1). The high scale level reflected the fact that the beluga whales were significantly brighter in the panchromatic image, compared to the open water and thus had distinct edges. The merge level of 40 was best since the beluga whales are not large whales at the resolution of the image (>3 m in a 31 cm resolution image), and therefore some, but limited, merging of objects into larger objects is required in the method. Classification of the merged objects to whales involved both spectral (mean) and texture (variance) attributes of the object (Table 1). The beluga whales even slightly below the surface, may appear brighter in the panchromatic image after a high-pass filter was applied; therefore, the utilization of the spectral mean and texture variance was able to classify the segments as whales versus open water. The addition of texture variance, greater than 1,500 for the high-pass filtered image, suggests that the spectral values for the whale objects in the high-pass filtered image did have some variation, which was used to classify these objects as whales.

Using the best algorithm (CA#10), the false positive rates of 0% for both the training and test tiles indicate that no objects in the images were misidentified as whales. The false negative rate suggests that the algorithm did misclassify a few whales identified by an experienced observer (#1) in the test image; however, this rate was very low (less than 4%). The false negative rate suggests that submerged or small whales (either in size of smaller area breaching the surface) may be misclassified as open water by the algorithm.

The use of this algorithm significantly reduced the time required to identify whales in the two tiles in excess of 72%. There was some variability in time required to identify the whales between the observers, especially in the training file that contains less whales overall. A researcher is still required to evaluate the output from the algorithm to determine a count estimate of the whales in the area; however, the use of this method will help locate the whales in the image, which can eliminate reading large areas of the imagery that contain no whales.

**Fig 5:**
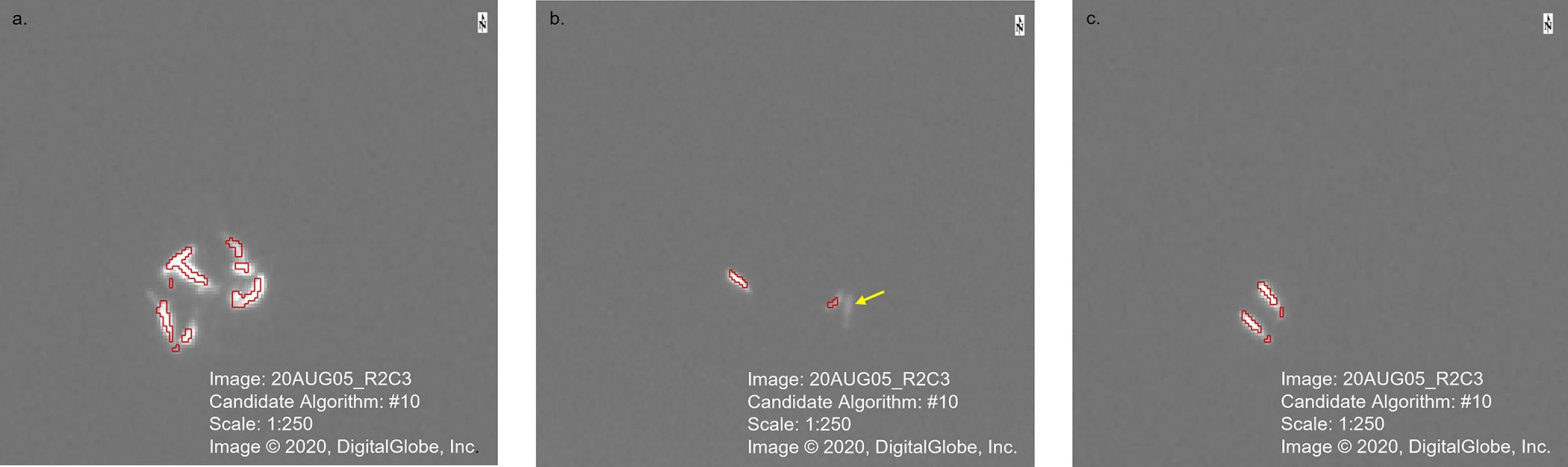
Examples of issues with the detection of beluga whales in the WorldView-3 satellite imagery: (a) grouped whales, (b) whales below the surface, and (c) multiple segments for each whale. Arrow indicates submerged whale in the image. (Reprinted under a CC BY license, with permission from Maxar Technologies, original copyright 2020).

There are some limitations to the use of the semi-automated object-based image analysis for identification of beluga whales. The algorithm was not able to separate whales that are in close proximity or in a group without open water between the whales (Fig 5A). For example, the segmentation is unable to distinguish two whales that are perpendicular to each other as two objects (Fig 5A), and thus identifies them as a single object. This is particularly a limiting factor for automating the counts of odontocetes, since many are known to travel in pods or herds with group sizes varying from small social units (three to ten individuals) [34], to intermediate-sized pods (ten to a few tens) [35], to large aggregations composed of hundreds or thousands of individuals [36, 37]. Beluga whales vary in group size from small groups of two to ten individuals to large herds of 2,000 or more individuals [38–40], and thus visual detection of these amalgamated segments will need to be done after the identification using the semi-automated detection. However, this may be less of an issue for using OBIA on large solitary baleen whales [41, 42]. One approach to rectify this limitation is to export the resulting shapefile into a GIS and separate features like this into two (or more) objects.

This method works well when there is significant contrast between the whale and the water in the image. However, the algorithm was not able to identify submerged whales since the spectral values are similar to those of open water and less similar to whales at the surface (Fig 5B). Varying the spectral mean value and/or texture measures, and the inclusion of other attributes in the classification stage of OBIA was not able to resolve this issue. Current research is trying to identify at which depth whales are no longer distinguishable in clear and turbid waters so that a correction factor may be applied to the final count, increasing the accuracy of population estimation. In addition, this algorithm may not work with species that are spectrally more similar to the water in the panchromatic image. Using a pansharpened image, combining the spectral information of multispectral imagery and the spatial resolution of a panchromatic image, may be able to resolve some of these issues.

A third limitation is that the OBIA method created multiple segments for individual whales (Fig 5C). The scale and merge levels identified the bodies and flukes of some of the whales as two separate objects. Adjusting these values in the algorithm would be able to combine these segments and for typically solitary whales, this is a good option; however, for whales that aggregate, this adjustment would also merge objects that represented two (or more) whales in close proximity, potentially increasing the ACD and decreasing the accuracy of the algorithm. Finally, the accuracy assessment of the various candidate algorithms is relative to the manual identification of the whales in both tiles, which is assumed to be correct. In this study, the variability in the whales identified by the two observers was low (2 whales in the training tile and 3 in the test tile), suggesting a high level of accuracy in the visual identification used for validation of the algorithm. However, any errors in this identification may lead to an overestimation of the accuracy of the algorithms.

In this paper, we showed the utilization of an object-based image analysis semi-automated method to identify whales in open water under ideal conditions (e.g., no sea ice, no cloud cover) in VHR satellite imagery. This automated process reduced the manual detection time by over 70% and can eliminate the need to review 100s of kilometers of whale-free imagery. Researchers reported varying amounts of time to manually process one km^2^ of satellite imagery. For example, Charry et al. [14] reported an approximate time of ~2.5 minutes at 1:536, Cubaynes et al. [16] reported an approximate time of 2 minutes at 1:1,500, and our study reported an approximate time ranging from five to eleven minutes to read one km^2^ at a scale of less than 1:800. Autodetection methods such as the one presented here offer a huge advantage in decreasing in the amount of time needed to process imagery as the candidate algorithms took on average 1.5 minutes to process one km^2^ of satellite imagery. In addition to reducing detection time this automated process is more consistent than manual imagery processing as manual observers did not have the exact same total count in our study and others [14]. The utility of using satellite imagery as a means for detecting whales across broad geographical regions can be realized with semi-automated detection, as the people power needed for manual detection is reduced significantly. This algorithm workflow can be adapted for detecting other whales across the world’s oceans. Future work is evaluating how to use multispectral and panchromatic imagery to identify whales in imagery with ice using a similar semi-automated object-based image analysis technique. This new method will be particularly useful for detecting whales in the Arctic in the spring and winter seasons, which are particularly logistically difficult to monitor without the use of satellite technology.

## Supporting information

S1 Table List of spectral (four), texture (four) and spatial (14) attributes used in Rule-Based Feature Extraction module in ENVI©. Descriptions of these attributes were obtained from ENVI© v.5.5 help files (https://www.l3harrisgeospatial.com/docs/attributelist.html#spatial_attributes).
(DOCX)
