# Supplementary material for: A semi-automated method using object-based image analysis (OBIA) to detect and enumerate beluga whales in summer from very high-resolution (VHR) satellite imagery
Source: PLoS One. 2024 Nov 13;19(11):e0307716. doi: 10.1371/journal.pone.0307716 (PMC11560058; doi:10.1371/journal.pone.0307716)
Supplement: S1 Table — Descriptions of these attributes were obtained from ENVI© v.5.5 help files (https://www.l3harrisgeospatial.com/docs/attributelist.html#spatial_attributes). (DOCX) [file pone.0307716.s001.docx]

**S1 Table. List of spectral (four), texture (four) and spatial (14) attributes used in Rule-Based Feature Extraction module in ENVI©. Descriptions of these attributes were obtained from ENVI© v.5.5 help files (**[**https://www.l3harrisgeospatial.com/docs/attributelist.html#spatial_attributes**](https://www.l3harrisgeospatial.com/docs/attributelist.html#spatial_attributes)**).**

| Attributes | | Description |
| --- | --- | --- |
| Spectral | Spectral_Mean | Average value of pixels for a segment for a particular band |
|  | Spectral_Max | Maximum value of pixels for a segment for a particular band |
|  | Spectral_Min | Minimum value of pixels for a segment for a particular band |
|  | Spectral_STD | Standard deviation of pixels for a segment for a particular band |
| Texture | Texture_Range | Range of pixels for a segment inside the kernel |
|  | Texture_Mean | Average spectral value of pixels for a segment inside the kernel |
|  | Texture_Variance | Variance of pixels for a segment inside the kernel |
|  | Texture_Entropy | Entropy of pixels for a segment inside the kernel |
| Spatial | Area | Total area of the segment, minus the area of the holes |
|  | Length | The combined length of all boundaries of the segment. |
|  | Compactness | The compactness of the segment |
|  | Convexity | The convexity of the segment |
|  | Solidity | Measure that compares the area of the segment to the area of the convex hull surrounding the segment |
|  | Roundness | Measure that compares the area of the segment to the square of the maximum diameter of the segment |
|  | Form_Factor | Compares the area of the segment to the square of the total perimeter |
|  | Elongation | Ratio of the major axis of the segment to the minor axis |
|  | Rectangular_Fit | Measure that indicates how well the shape is described by a rectangle |
|  | Main_Direction | Angle subtended by the major axis of the segment and the x-axis (in degrees) |
|  | Major_Length | Length of the major axis of the segment |
|  | Minor_Length | Length of the minor axis of the segment |
|  | Number_of_Holes | Number of holes in the segment (dependent on the kernel size) |
|  | Hole_Area/Solid_Area | Ratio of the total area of the segment to the area of the outer contour. |
